# Interference of unilateral lower limb amputation on motor imagery rhythm and remodeling of sensorimotor areas

**DOI:** 10.3389/fnhum.2022.1011463

**Published:** 2022-11-03

**Authors:** Shaowen Liu, Wenjin Fu, Conghui Wei, Fengling Ma, Nanyi Cui, Xinying Shan, Yan Zhang

**Affiliations:** ^1^Department of Neurosurgery, The Second Affiliated Hospital of Nanchang University, Nanchang, Jiangxi, China; ^2^Beijing Key Laboratory of Rehabilitation Technical Aids for Old-Age Disability, National Research Center for Rehabilitation Technical Aids, Beijing, China; ^3^Brainup Institute of Science and Technology, Chongqing, China

**Keywords:** motor imagery, amputation, phantom limb pain, time-frequency analysis, neuroplasticity

## Abstract

**Purpose:**

The effect of sensorimotor stripping on neuroplasticity and motor imagery capacity is unknown, and the physiological mechanisms of post-amputation phantom limb pain (PLP) illness remain to be investigated.

**Materials and methods:**

In this study, an electroencephalogram (EEG)-based event-related (de)synchronization (ERD/ERS) analysis was conducted using a bilateral lower limb motor imagery (MI) paradigm. The differences in the execution of motor imagery tasks between left lower limb amputations and healthy controls were explored, and a correlation analysis was calculated between level of phantom limb pain and ERD/ERS.

**Results:**

The multiple frequency bands showed a significant ERD phenomenon when the healthy control group performed the motor imagery task, whereas amputees showed significant ERS phenomena in mu band. Phantom limb pain in amputees was negatively correlated with bilateral sensorimotor areas electrode powers.

**Conclusion:**

Sensorimotor abnormalities reduce neural activity in the sensorimotor cortex, while the motor imagination of the intact limb is diminished. In addition, phantom limb pain may lead to over-activation of sensorimotor areas, affecting bilateral sensorimotor area remodeling.

## Introduction

The human brain is a complex and dynamic system whose structure and functions are dynamically shaped in the course of development (Fries, 2005; Kang et al., 2011). Structural changes and functional reorganization of the cerebral cortex take place as a result of learning, adapting to disease, exposure to particular environment, and this neural ability to adapt to new reality and sustain functionality is known as neuroplasticity (Jaberzadeh and Zoghi, 2013; Cantone et al., 2020). Neuroplasticity has been illustrated in patients of stroke, amputation and spinal cord injury (Kolb et al., 2014; von Bernhardi et al., 2017). In the case of amputation, complete and abrupt deprivation of all afferent and efferent nerves of the amputated limb from the central nervous system causes network reorganization in the sensorimotor cortex.

The properties of neuroplasticity manifests in structural and functional changes after amputation. In the primary somatosensory cortex (SI), sensory deprivation leads to recruitment of the input-deprived cortex by neighboring cortical representations, resulting in massive structural (Florence et al., 1998) and functional (Pons et al., 1991) areal cortical reorganization. Amputation not only led to a reorganization of the cortical representation area of the missing hand, but also reduced the functional connectivity between the aforementioned deprived hand region and the sensorimotor network, and the strength of the functional connectivity diminished with time after amputation (Makin et al., 2015). Animal studies have shown that the loss of part of a limb leads to a functional reorganization of the sensorimotor cortex (Florence and Kaas, 1995; Jain et al., 2000). Similar results have been seen in human studies, where upper or lower amputation have been associated with remodeling of cortical (Zhang et al., 2018) and subcortical (Draganski et al., 2006) brain function and structure, with different sites of amputation producing different results. Patients with upper limb amputations showed generalized brain remodeling in sensory and motor areas (Flor et al., 2013). People born with one hand whose brains have plastically adapted to the missing hand early on did not suffer phantom limb pain. Striem-Amit’s hypothesized, in the absence of pain, plasticity manifests as a compensatory mechanism notably replaced by foot or mouth functional representative areas (Striem-Amit, 2017).

The amputee will feel the presence of the missing limb and can feel specific sensations (phantom limb sensation) and/or pain (phantom limb pain) on the phantom limb after amputation/blocking of the limb. Cortical reorganization in patients with phantom limb pain involve more multidimensional cognitive alterations such as pain and emotion, which may result in more complex patterns of brain reorganization, the pathophysiological mechanisms of which are still unclear and need further study. There is evidence that phantom limb pain may be a central nervous system phenomenon associated with plasticity changes in the cerebral cortex (Flor et al., 2006). Amputees with phantom limb pain showed a significant increase in bilateral motor cortex activation during intact hand movements, especially in the cerebral cortex corresponding to the missing limb, suggesting a non-adaptive reorganization of the cerebral cortex (Bogdanov et al., 2012). Mouth functional representative areas and hand functional representative areas were activated during lip movements in patients with phantom limb pain, and the corresponding cortical activity was more pronounced during imagined phantom limb activity than during intact hand activity (Lotze et al., 2001). The primary sensory cortex (S1) and the primary motor cortex (M1) were reorganized in patients with phantom limb pain, and their activation areas expanded from the mouth representative area to the hand sensory representative area during lip movements, and the distance of their expansion was proportional to the pain index (MacIver et al., 2008). It is suggested that the absence of sensory input leads to non-adaptive reorganization of the cerebral cortex and has been shown to correlate with the degree of phantom limb pain. Phantom pain in amputees is associated with reduced interregional functional connectivity in primary sensorimotor cortices, and chronic phantom limb pain experiences drive plasticity by maintaining local cortical expression and disrupting interregional connectivity (Makin et al., 2013). It is noteworthy that motor imagery was not disabled by unilateral limb loss in amputees. Loss of a single limb does not prevent motor imagery but it significantly enhances its difficulty (Nico et al., 2004). Task-phantom vividness was significantly positively correlated with response time and the rotation-related beta-ERD, which suggest that phantom limb perception is an important interference factor in motor imagery after amputation (Lyu et al., 2016). This study aimed to investigate the changes in motor pathways and brain plasticity in amputees by studying brain activity in the motor imagery state.

Subjects were not required to participate in any specific task in the resting state, but amputees were not always in rest-state. Lower limb amputee (LLA) who has severely impaired motor function due to lack of lower extremities while having normal cognitive function are able to perform motor imagery tasks. Benefiting from MI, cortex-lesioned patients exhibit better functional recovery (Page et al., 2001), and BCI users obtain higher BCI classification accuracy (deCharms, 2007). Due to the high temporal resolution, low cost, and easy availability, comparing to the functional magnetic resonance imaging (fMRI), electroencephalogram (EEG) is widely used in related MI studies (Zhang et al., 2015). For the motor imagery tasks, the participants were asked to imagine moving their limb without producing actual movement. The primary characteristics of MI-EEG is event-related desynchronization (ERD) or event-related synchronization (ERS), representing the decrease, or increase of the EEG power, respectively over the sensorimotor cortex, generally in the mu and beta rhythms (Pfurtscheller et al., 2006). Performing motor imagery also requires the activation of the corresponding motor cortex of the relevant limb. Motor imagery and motor execution share a similar pattern in that the brain areas that need to be activated to perform both activities are highly overlapping (Lotze and Halsband, 2006), for example the contralateral BA4, premotor cortex (PMc), parietal areas, and supplemental motor area (SMA) are activated areas during motor imagery (Sharma and Baron, 2013). The magnitude of changes in cortical activity induced by motor imagery is 25% of that of motor execution (ME). Mental imagery practice process can be used as a preview of actual activities to complete the learning of simple to complex motor skills (Schekman, 2010). Motor imagery also has a wide range of applications in the field of neurorehabilitation and brain-computer interface (BCI), and is important for restoring function in patients with motor dysfunction (e.g., amputations, strokes, etc.) (Lotze and Cohen, 2006; Li et al., 2010, 2016; Burianova et al., 2013). Amputees who are unable to perform motor training can benefit from motor imagery therapy.

The limitations of the existing studies in this field are mainly that the patients have varying degree of amputation and on different sides. Many studies have included patients with amputation on both sides, and performed analysis without taking contralateral bias into account. In the nervous system, brain areas associated with sensorimotor function are symmetrical and follow a contralateral projection pattern. Therefore, the effect of brain remodeling induced by unilateral amputation patients on motor imagery is well worth investigating. In addition, the altered neuroplasticity of phantom limb pain on primary somatosensory cortex is also worth investigating. Left lower limb amputees were recruited as subjects in this study to explore the effect of phantom limb pain on the execution of motor imagery tasks in amputees under specific rhythms. The investigation of abnormal sensation and nociception associated with lower limb amputation helps to understand the neural mechanisms of brain function remodeling, which is conducive to the development of new rehabilitation methods, while providing a theoretical basis for the development of intelligent prostheses to improve the quality of life of lower limb amputee patients.

## Materials and methods

### Participants

Written informed consent was obtained from each participant under Ethics Committee of the National Research Center for Rehabilitation Technical Aids approved protocols. Key eligibility for the study included the following: being 18–75 years old; having no cognitive impairment; left unilateral lower limb amputation; right-hand dominant. Exclusion criteria included the following: lifetime history of psychosis or bipolar disorder; presence of major systemic disease (example: stroke, coronary heart disease, etc.); and the patient was unable to perform the experiment due to severe phantom limb pain. A total of 19 Participants (one female) with acquired unilateral left lower limb amputation (mean age ± sd = 51.21 ± 12.98, see Table 1) were recruited through the Rehabilitation Hospital Affiliated to National Research Center for Rehabilitation Technical Aids. A total of 22 healthy controls (three females), matched for age (mean age ± sd = 45.41 ± 9.45; t = 1.561, *p* = 0.107) and handedness (zero left-hand dominant) were also recruited. Each subject was informed of the purpose of the experiment, the method of study, and that the experiment was completely free of any damage to the brain.

**TABLE 1:** Demographic and clinical details of the amputees.

ID	Age (years)	M/F	Amputation location	Time since amputation	Cause for amputation	PLP-frequency	RLP–frequency
1	32	M	Left shank	4 months	Accident	6/2	0
2	45	M	Left shank	5 months	Accident	0	0
3	28	M	Left thigh	4 months	Accident	3/3	0
4	41	M	Left thigh	10 months	Accident	5/2	2/3
5	52	M	Left thigh	3 months	Congenital clubfoot	4/2	0
6	53	M	Left thigh	4 months	Burn	3/1	3/3
7	49	M	Left thigh	2 months	Accident	3/2	0
8	46	M	Left thigh	4 months	Accident	9/1	9/2
9	41	M	Left shank	8 months	Accident	5/2	2/3
10	66	F	Left thigh	11 months	Accident	4/2	0
11	45	M	Left shank	4 years	Accident	1/3	4/3
12	50	M	Left thigh	8 years	Accident	6/2	0
13	52	M	Left shank	18 years	Accident	0	0
14	74	M	Left shank	38 years	Accident	0	0
15	67	M	Left shank	24 years	Accident	0	0
16	72	M	Left shank	30 years	Accident	2/2	2/2
17	46	M	Left shank	2 years	Accident	4/2	3/3
18	69	M	Left shank	38 years	Accident	0	0
19	45	M	Left shank	4 years	Accident	0	0

PLP, phantom limb pain; RLP, residual limb pain; M, male; F, female.

### Behavioral assessment

Clinical data (Table 1) related to the amputation as well as phantom limb pain (PLP) residual limb pain (RLP) were collected before the MI-task. Amputees rated the frequencies of phantom/stump pain, as experienced within the last year, as well as intensity of worst pain experienced during the last week (or in a typical week involving phantom/stump sensations). “Pain magnitude” was calculated by dividing pain intensity (0: “no pain”—10: “worst pain imaginable”) by frequency (1—“all the time,” 2—“daily,” 3—“weekly,” 4—“several times per month,” and 5—“once or less per month”). This measure therefore reflects the chronic aspect of the pain as it combines both frequency and intensity, as used previously (Makin et al., 2015).

### Experimental paradigm

**FIGURE 1:**
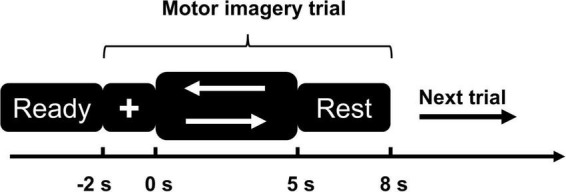
Motor imagery (MI) paradigm diagram. The left arrow represents the left-side MI task; the right arrow represents the right-side MI task.

During the motor imagery experiment, the subject sat on a chair and randomly executed left or right leg extension task, but did not produce the actual movement (Kitahara et al., 2017). We used a bilateral leg task to compare and analyze the differences in neuronal responses elicited by intact limb MI and missing limb MI (Raffin et al., 2012). The experimental procedures of the MI-task is shown in Figure 1. Stimulus presentation was controlled by E-prime 2.0 experimental software (Psychology Software Tools, Inc., Sharpsburg, PA, USA). This experimental paradigm consisted of 120 trials (performing left/right side MI), each trial lasted 10 s. Participants first practiced the tasks, then completed two blocks of 60 trials (120 trials total), with a 5 min break between each block, and a total task duration of 25 min. The trial steps were as follows:

1) First step was a 3 s “Ready” phase, the screen shows black background.
2) After the “Ready” phase, a white cross appeared on the screen with black background and lasted for 2 s, during which time the subjects were asked to focus their attention.
3) Visual cues (white left or right arrows, selected randomly) appeared on the screen and lasted for 5 s. Subjects performed left or right MI task but did not actually make any movement.
4) Subjects rested for 3 s and then proceeded to the next trial.

### Electroencephalogram acquisition

In this experiment, the subjects completed the whole process in the EEG acquisition room of the National Research Center for Rehabilitation Technical Aids, and the 256-channel EEG acquisition system of Net Station Acquisition EGI dense array EEG was used to record EEG data without involving other equipment. The instrument used for EEG signal acquisition was the 256-channel EGI system, including the EEG cap, the amplifier, and the signal acquisition software. To ensure good quality EEG signals, participants were asked to wash their hair before attending the recording session. After the EEG equipment was set up, the subjects sat in a comfortable chair and began EEG data acquisition for 25 min. EEG was recorded with electrode impedances ≤50 kΩ, a sampling frequency of 500 Hz, and with Cz as reference electrode.

### Electroencephalogram data preprocessing

The raw EEG data were preprocessed using EEGLAB (v.14.0.0b) (Delorme and Makeig, 2004) offline on Matlab (The Math Works Inc., Natick, MA, USA) software. The bandpass filter was set at 0.1–30 Hz using EEGLAB\FIR. EEG data were re-referenced with a whole brain average reference. Epochs were extracted from –2 to 5 s relative to arrow onsets. Bad EEG segments (those exceeding ±200 μV in any channel) and artifacts were rejected manually. The EEG data were manually checked for interpolation for bad electrodes. Independent component analysis (ICA) was used to identify and remove residual artifacts such as blinks, lateral eye movement and voltage drifts.

## Time frequency analysis

### Electroencephalogram analyses

We used the EEGLAB toolbox in the MATLAB environment to perform time-frequency analysis of the EEG data after preprocessing. The specific mathematical principles and details were elaborated in a previous study (Delorme and Makeig, 2004). In this study, time-frequency analysis was performed using the Fourier transform method to analyze EEG data in the frequency range of 1–30 Hz and the time range of –2,000 to 5,000 ms. To ensure balanced time and frequency resolution, we set the time resolution to 35 ms and the frequency resolution to 0.9 Hz. The epoch EEG data of 19 amputees and 22 healthy controls were averaged separately.

### Statistical analyses

**FIGURE 2:**
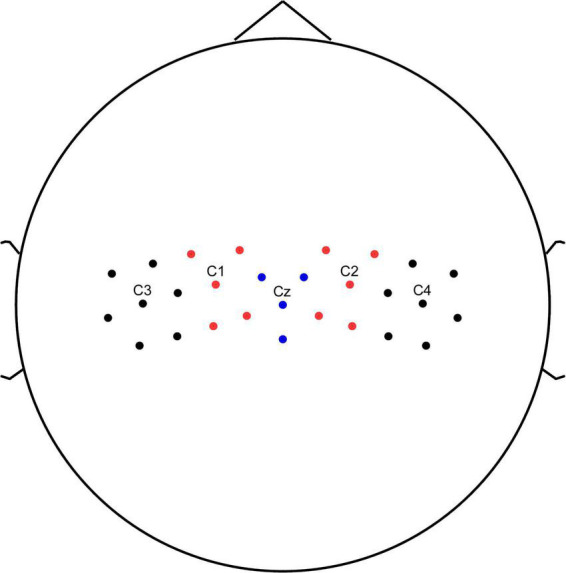
Channel location. C3, C1, Cz, C2, and C4 electrodes and multiple surrounding electrodes into five representative electrodes.

Time-frequency analysis of the EEG data were conducted using event-related spectral perturbation (ERSP) analysis for a 5 s window that extended from 0 to 5 s relative to arrow onset. We used the sensorimotor region of the brain as the target brain area for the study, and C3, C1, Cz, C2, and C4 as the main electrodes for the study. We averaged the C3, C1, Cz, C2, and C4 electrodes and multiple surrounding electrodes into five representative electrodes (Figure 2) to obtain the average event-related spectral perturbation (ERSP) (relative to baseline levels) for each of the two groups.

Statistical analyses were performed with the SPSS software, Version22 (IBM Corp., New York, NY, USA). We analyzed the differences in the magnitude of event-related spectral perturbation for five electrodes (C1, C2, C3, C4, CZ) in the five frequency bands (delta 1–4 Hz, theta 4–7 Hz, mu 8–12 Hz, lower beta 13–20 Hz, high beta 21–30 Hz) between amputees and healthy controls using one way ANOVA.

Finally, we assessed the correlation between the phantom limb awareness and motor imagery task of the missing limb by correlation analysis. We collected PLP and RLP scores from 19 patients with left-sided amputations and correlated indicators extracted from EEG data analysis with PLP/RLP using Pearson’s correlation method.

## Results

### Behavioral results

Among amputation patients, phantom limb pain and residual limb pain were present in 13 and seven patients, respectively. Phantom limb pain (r = –0.558, *p* = 0.013) decreased significantly with the presence of increasing amputation time length. There was no significant difference between the residual limb pain and the time length of amputation (r = –0.245, *p* = 0.313). There was no significant difference between the residual limb pain and the time length of amputation (r = –0.245, *p* = 0.313). Age was not significantly correlated with either PLP (r = –0.443, *p* = 0.064) or RLP (r = –0.152, *p* = 0.534).

### Time-frequency analysis results

**FIGURE 3:**
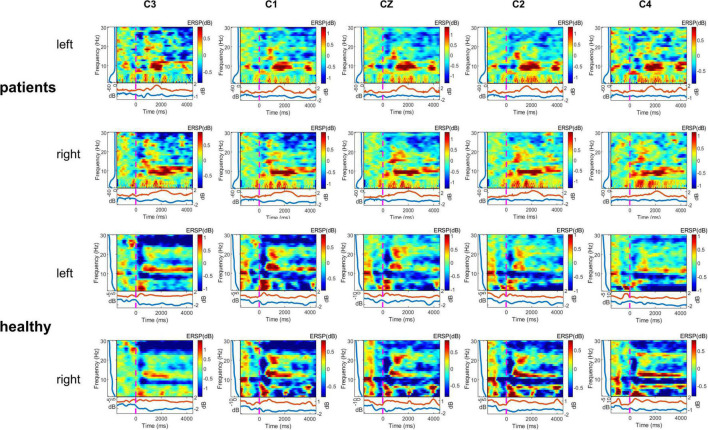
Time—frequency maps displaying significant event-related desynchronization (ERD) (blue) and event-related synchronization (ERS) (red), two motor imagery (MI) (left and right) task, two group (lower limb amputees and healthy controls), and five channels (C3, C1, Cz, C2, C4). Maps displaying group averaged power (ERD, ERS) in the 1–30 Hz band during two motor imagery tasks (second –2 to 5 s of the trial). Left, left-side MI task; right, right-side MI task.

As shown in Figure 3, the multiple frequency bands showed a significant ERD phenomenon when the healthy control group performed the motor imagery task. Lower limb amputation patients showed a significant ERS phenomenon in mu rhythm when performing the motor imagery task.

**FIGURE 4:**
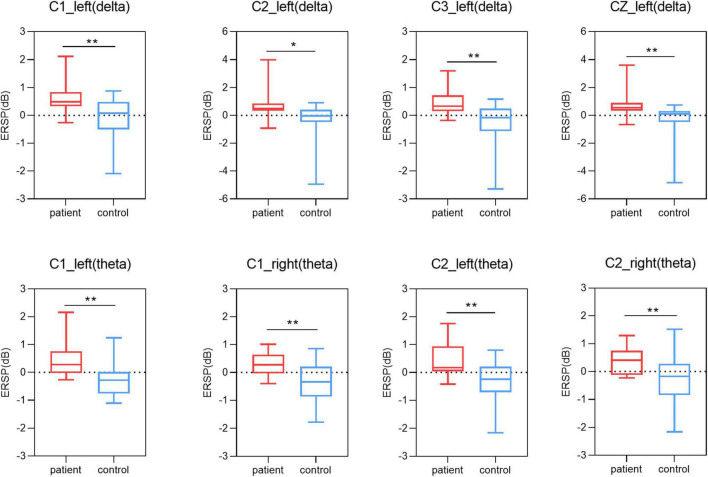
Results of one-way ANOVA. Comparison of power in delta and theta band and five channels (C3, C1, Cz, C2, C4) during two motor imagery (MI) tasks (second 0–5 s of the trial) among lower limb amputees and healthy controls. Left, left-side MI task; right, right-side MI task; *p < 0.05; **p < 0.01.

**FIGURE 5:**
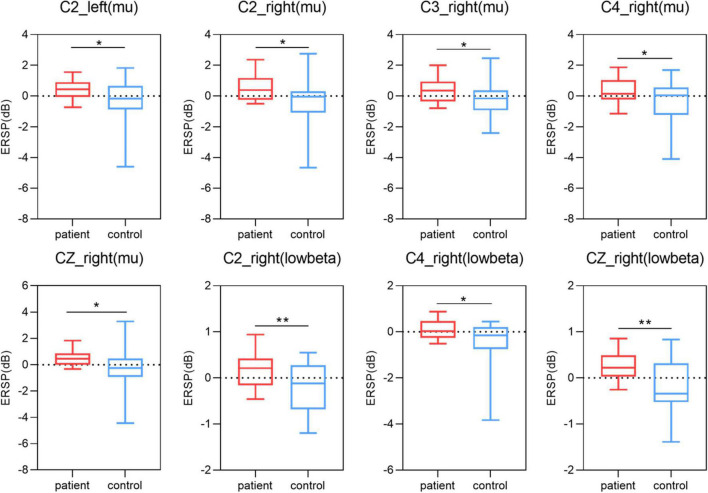
Results of one-way ANOVA. Comparison of power in mu and low beta band and five channels (C3, C1, Cz, C2, C4) during two motor imagery (MI) tasks (second 0–5 s of the trial) among lower limb amputees and healthy controls. Left, left-side MI task; right, right-side MI task; *p < 0.05; **p < 0.01.

In this study, we used One-way ANOVA to compare the difference in power between lower limb amputees and healthy controls in five frequency bands on sensorimotor area electrodes (C3/C1/Cz/C2/C4). When LLAs performing the task condition of left side MI, we found some differences in the delta band (F = 12.481, 10.756, 7.769, 7.202, 3.298; *p* = 0.001, 0.002, 0.008, 0.011, 0.077) (Figure 4), theta band (F = 13.141, 13.217, 9.251, 12.504, 5.756; *p* = 0.001, 0.001, 0.004, 0.001, 0.021) (Figure 4), mu band (F = 2.204, 2.946, 3.200, 4.778, 3.487; *p* = 0.146, 0.094, 0.081, 0.035, 0.069) (Figure 5), but not in the low beta (F = 1.074, 0.951, 2.061, 1.479, 0.058; *p* = 0.306, 0.335, 0.159, 0.231, 0.811) and high beta bands (F = 0.166, 0.031, 0.000, 0.154, 0.097; *p* = 0.686, 0.861, 0.989, 0.697, 0.758).

When LLAs performing the task condition of right side MI, we found some differences in the theta band (F = 6.204, 11.599, 11.389, 8.936, 3.430; *p* = 0.017, 0.002, 0.002, 0.005, 0.072) (Figure 4), mu band (F = 5.010, 2.727, 4.423, 4.236, 4.592; *p* = 0.031, 0.107, 0.042, 0.046, 0.038) (Figure 5), low beta (F = 1.952, 1.193, 7.713, 7.527, 4.857; *p* = 0.170, 0.281, 0.008, 0.009, 0.034) (Figure 5), but not in the delta band (F = 0.161, 2.747, 2.778, 2.665, 0.661; *p* = 0.691, 0.105, 0.104, 0.111, 0.077) and high beta bands (F = 0.729, 0.059, 0.489, 0.684, 0.209; *p* = 0.398, 0.810, 0.488, 0.413, 0.650).

### Correlation analysis results

**FIGURE 6:**
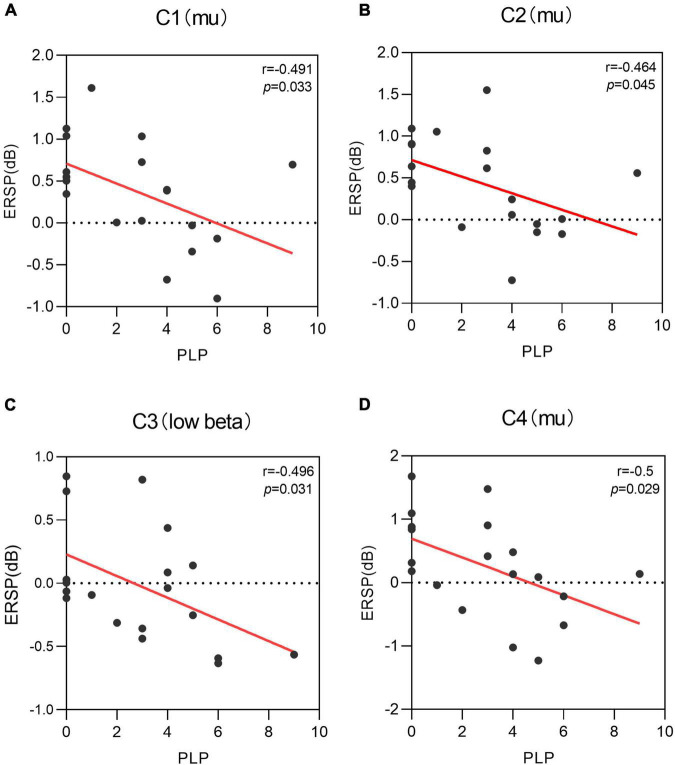
Correlation of phantom limb pain (PLP) with the power of different electrodes when performing the left motor imagery task, i.e., there is a negative correlation between PLP and the power of multiple electrodes. **(A)** A negative correlation was found between C1 power values in mu rhythm and PLP level (r = –0.491, *p* = 0.033). **(B)** A negative correlation was found between C2 power values in mu rhythm and PLP level (r = –0.464, *p* = 0.045). **(C)** A negative correlation was found between C3 power values in low beta rhythm and PLP level (r = –0.496, *p* = 0.031). **(D)** A negative correlation was found between C4 power values in mu rhythm and PLP level (r = –0.5, *p* = 0.029).

The present study used Pearson correlation analysis for correlation and found a significant negative correlation between PLP (r = –0.491, *p* = 0.033) and mu rhythm power at C1 electrodes in the left side MI task condition (see Figure 6A). A significant negative correlation between PLP (r = –0.464, *p* = 0.045) and mu rhythm power at C2 electrodes (see Figure 6B). A significant negative correlation between PLP (r = –0.496, *p* = 0.031) and low beta rhythm power at C3 electrodes (see Figure 6C). A significant negative correlation between PLP (r = –0.5, *p* = 0.029) and mu rhythm EEG power at C4 electrodes (see Figure 6D). Neither delta band (r = –0.129, –0.219, –0.416, –0.080, 0.007; *p* = 0.599, 0.369, 0.076, 0.743, 0.977) nor theta band (r = –0.0229, –0.219, –0.160, –0.133, –0.055; *p* = 0.345, 0.369, 0.512, 0.586, 0.822) on the electrodes (C3, C1, Cz, C2, C4) in the sensorimotor area was significantly correlated with PLP.

## Discussion

In the present study, we investigated the effect of unilateral lower limb amputation on motor imagery. There was significant increase in the power of patients with lower limb amputation compared to healthy controls at multiple frequency band (delta, theta, mu, and low beta) when performing the both side MI task, i.e., the absence of the limb affect MI. PLP scores in amputees were negatively correlated with the power of mu and low beta revealing that phantom limb awareness is an important factor affecting MI ability. In addition, the time length of amputation was significantly and negatively correlated with phantom limb pain, and there was a corresponding adaptation of the organism to pain.

A clear image of an intended action can be present even without the limb involved in movement execution as it has been demonstrated in patients after traumatic limb amputation (Raffin et al., 2012) or deafferentation of half of the body after complete thoracic spinal cord injury (Decety and Boisson, 1990). During the process of performing different MI tasks, the metabolism and blood flow in that area increases and the simultaneous processing of information can lead to a reduction in the amplitude of the alpha and beta frequency bands of brain waves or a blockage (Flor et al., 2006). After MI task presentation, ERDs are evident in the mu rhythm (Pfurtscheller et al., 2006). Lower limb amputation leads to significant neural reorganization within the central nervous system (CNS) mostly due to the loss of the sensorimotor function caused by amputation (Geurts et al., 1991; Chen et al., 1998). Previous studies have demonstrated that amputees can imagine the missing limb (Reilly et al., 2006). Amputees showed reduced vividness during MI of the limb but were still able to perform the task, especially during the visual-MI scale (Kinesthetic and Visual Imagery Questionnaire, KVIQ) scores during imagery of the missing limb were lower than the KVIQ scores of the intact limb (Malouin et al., 2009). The functional non-use of the missing-hand region in upper limb amputees leads to reduction of gray matter in the motor representation zone of this hand (Preissler et al., 2013). Motor imagery elicits ERD production in sensorimotor areas requiring large motor neuron activation, but amputees producing ERS. Amputees performing bilateral lower limb MI tasks exhibit predominantly ERS in the mu band, which may be due to the inability of neurons to perform the task synchronously and the corresponding decrease in neural activity.

When lower limb amputees performing the task condition of left side motor imagery, our results revealed that there was a significant increase in delta, theta and mu bands power for missing limb electrodes compared to the healthy controls. The missing limb electrode of the left lower limb amputees produced an ERS phenomenon, indicating a marked decrease in the ability of the reorganized cortex to perform motor imagery. Following amputation, the region that represented the missing limb in primary somatosensory cortex (S1) becomes deprived of its primary input, resulting in changed boundaries of the S1 body map (Makin and Flor, 2020). Reorganization of the primary somatosensory cortex was associated with increased chronic phantom limb pain, telescoping, non-painful stump sensations, and painful referred sensation induced by painful stimulation (Grusser et al., 2001). When lower limb amputees performing the task condition of right side motor imagery, our results revealed that there was a significant increase in theta and mu band power for intact limb electrodes compared to the healthy controls. FMRI study shows decreased functional connectivity of primary sensorimotor cortex to thalamus and basal ganglia (Zhang et al., 2018). Researcher found a pronounced reduction of inter-hemispheric functional connectivity between bilateral sensorimotor cortical regions in amputees, including the primary (S1) and secondary (S2) somatosensory areas, and primary (M1) and secondary (M2) motor areas (Bramati et al., 2019). Similar results are reflected in the enlarged cortical activation areas and diminished interhemispheric functional connectivity detected in both primary sensorimotor cortex when studying functional connectivity in amputated brains (Gong et al., 2009; Power et al., 2011), a phenomenon that has been interpreted as a maladaptive manifestation of the recovery process of the body in response to dysfunction triggered by impairments in sensory input or autonomic control. This phenomenon on fMRI is reduced functional connectivity between the cerebral hemispheres, which may reflect a chronic lack of synergistic activation between the cortices of the intact and missing limbs (Makin et al., 2013). Amputation leads not only to structural reorganization of the cortex of the missing limb, but also to functional changes in the cortex of the intact limb, which is reflected in increased power in the EEG and manifested as ERS in the mu bands. Alpha rhythm (8–13 Hz) is one of the main components of spontaneous EEG, alpha waves are generated throughout the cerebral cortex, and are most pronounced in the occipital lobe. Mu rhythms are EEG recorded in sensorimotor areas and are distinguished from alpha rhythms in that mu rhythms are not influenced by vision but change due to movement, movement preparation or motor imagery (Lotze and Halsband, 2006), hence mu rhythms are also known as sensorimotor rhythms. Amputation is a strong driver of neuro plasticity, and after lower limb amputation the cerebral cortex lacks corresponding sensory-motor and limb-visual inputs to the lower limb, while maintaining muscle memory in the brain’s sensorimotor cortex depends on limb sensorimotor and visual inputs (Flor et al., 2006). Amputees exhibit an increased need to use visual information to perceive objects due to the absence of limb sensory input, showing increased gray matter in brain regions with dorsal and ventral visual streams (Preissler et al., 2013). This enhanced need for visual information will probably manifest itself as an increase in occipital gray matter, leading to a decrease in the power of the alpha rhythm.

Patients with limb amputation usually show vivid representation of the arm and leg even many years after deafferentation (Makin et al., 2013). In the present study, the level of PLP was significantly and negatively correlated with ERD in mu and low beta rhythms but not with delta and theta rhythms, showing that that sensorimotor rhythms are important for the study of phantom limb pain. The presence of phantom limb awareness is associated with enhanced MI brain activity, which reveals a brain remodeling mechanism for phantom limb awareness. This is similar to previous findings that phantom limb pain may lead to over-activation of motor areas (MacIver et al., 2008). Amputees maybe performed movement of the phantom hand rather than imagined movements, it is likely that phantom movements in amputees substantially activate both primary motor cortex (cM1) (Ersland et al., 1996). During MI of the phantom hand all 14 amputees included showed increased activation in both cM1 and cS1 compared to healthy controls (Lotze et al., 2001). Amputees with phantom limb awareness perform MI tasks more similar to motor execution, and this similarity is not conducive to distinguishing between the two tasks and is a challenge for the development of motor imagery-based BCI devices.

Unilateral lower limb loss does not cause loss of MI in patients with phantom limb pain. This phenomenon is an adaptive and protective mechanism of the organism to abnormal sensory input and also provides an electrophysiological basis for the disturbing effect of phantom limb pain on MI. During the execution of a MI task, amputees with phantom limb sensation had significantly longer reaction times and execution times compared to healthy controls, while amputees without phantom sensation had no significant prolongation (Lyu et al., 2016). In addition, the β-ERD activity was diminished in the group with phantom limb sensation, ERD activity can reflect cortical activity in the frequency domain, and the most basic reason for the reduced activation in local brain areas is that the reduced processing time allows for a reduced number of neuronal activations, which may reflect a more rapid processing of relevant information by specific neural networks. Amputees lose their original sensory input and develop abnormal phantom limb pain, which causes changes in specific brain regions. That the chronic PLP experience, which may be triggered either by bottom-up nociceptive inputs or top-down inputs from pain-related brain areas, drives plasticity because it maintains local cortical representations and disrupts inter-regional connectivity (Makin et al., 2013). In the present study, the phantom limb pain was correlated not only with the missing limb representative cortical electrodes (C2/C4 power, Figures 6B,D) but also with the intact limb representative cortical electrodes (C1/C3 power, Figures 6A,C). Phantom limb pain in the missing limb does not damage only the contralateral sensorimotor area, but also the ipsilateral intact cortical representation area. In addition to sensorimotor areas, some researchers have reported changes in pain-related brain areas, such as insula and anterior cingulate, and prefrontal cortex volumes in patients with phantom limb pain, predicting structural brain remodeling in pain-related brain areas in phantom limb pain as well (Li et al., 2017). Previous studies found that the activation of the inferior parietal lobule was significantly enhanced when tactile stimulation induced the development of phantom limb sensation in amputees, and it was hypothesized that the inferior parietal lobule was associated with the development of phantom limb sensation after amputation (Flor et al., 2000). In patients with phantom limb pain, phantom limb pain was significantly relieved when sensory phantom limb and virtual mirror limb congruency increased, and inferior parietal lobule activation decreased during mirror therapy (Foell et al., 2014). Therefore, phantom limb pain is capable of interfering with MI after amputation, and the phantom limb experience makes an important factor that cannot be ignored when studying MI in amputees in the future.

Lower limb amputees maintain normal cognitive function and are able to perform MI tasks, but the remodeling of brain function associated with phantom limb pain after amputation cannot be ignored. Phantom hand movement-evoked activity in sensory-motor deficient hand cortex is negatively correlated with PLP (Kikkert et al., 2018). People who experience more chronic PLP also show greater activity when moving the phantom hand (Makin et al., 2015). This coincides with the negative correlation between the degree of phantom limb pain and the cortical electrodes of the missing and intact limbs in this study, meaning that phantom limb pain over-activates the relevant cortex. Phantom limb pain in amputees is closely related to cortical remodeling, but unfortunately the current methods available to treat chronic phantom limb pain are far from satisfactory (Nikolajsen et al., 2013). Analysis of EEG rhythms in amputees under a motor imagery task revealed that PLP was correlated with mu and beta rhythms. Correlation of sensorimotor rhythms with phantom limb pain provides EEG evidence to explore the mechanism of phantom limb pain. Motor imagery-based EEG maybe used as an assessment tool for the treatment of phantom limb pain.

In present study, age was not significantly correlated with either PLP/RLP. Phantom limb pain seems to be equally frequent in traumatic and medical amputees, and age, sex, side, and level of amputation have been reported not to affect the prevalence of pain (Nikolajsen and Christensen, 2015). We derived similar results to previous studies. In present study, LLAs showed a decrease in phantom limb pain as the duration of amputation increased. Gray matter decreases of the posterolateral thalamus contralateral to the side of the amputation were positively correlated with the time span after the amputation (Draganski et al., 2006). The fMRI results showed a decrease in gray matter thickness in the middle temporal lobe of the visual area, and the longer the duration of amputation, the smaller the gray matter thickness in this brain area (Jiang et al., 2016). The time since amputation may be a more important influence for neuroplasticity than age.

Patients of different amputation ages have many differences in brain remodeling. Amputees without phantom pain are usually amputated at an earlier age than those with phantom pain (Diers et al., 2010). Congenital amputees do not exhibit phantom limb pain and show compensatory remodeling, but phantom limb pain is positively correlated with cortical reorganization in traumatic amputees (Flor et al., 1998). Patients with congenital unilateral handedness use multiple body parts to compensate for their disability, and body parts that replace hand function are remapped to brain regions of the missing hand (Hahamy et al., 2017). The brain of congenital one-handed individuals is already plastically adapted to the absence of one hand from early development, when the brain is most plastic (Striem-Amit, 2017). Importantly, this cortical reorganization was not accompanied by phantom pain, suggesting that the plasticity process in congenital amputees may be compensatory rather than maladaptive. There are fewer articles about how age at the time of amputation affects brain plasticity. In the future, we can include patients of different ages but with similar time of amputation in the study.

## Conclusion

Both MI and motor execution apparatus activate relevant brain regions, and the ERD phenomenon is evident in multiple frequency band in healthy individuals after stimulus presentation, while it is not seen in patients with lower limb amputation. Amputation is a strong driver of neuroplasticity, and the absence of significant ERD in the mu bands during MI is an EEG manifestation of structural changes as well as functional reorganization in the cerebral cortex after amputation. In addition to the remodeling of the brain region contralateral to the missing limb, remodeling also occurred in the ipsilateral brain region. Sensorimotor rhythms are closely related to PLP, providing a basis for exploring phantom limb pain and cortical reorganization. PLP may lead to over-activation of sensorimotor areas and correlated with amputation duration, reflecting adaptive changes in the brain after amputation.

### Limitations and outlook

This paper did not include prosthetic wear in the study, and the correlation between the phantom limb pain and brain remodeling and prosthetic wear remains poorly studied. The patients with amputation recruited in this paper spanned a wide range of amputation durations, and subsequent studies could be explored in multiple groups by duration, including acute, recovery, and chronic phases. A large number of smart prostheses based on MI are being developed in the future, and brain remodeling from amputation and phantom limb pain should be included in the study of smart prostheses. Patients should be evaluated for phantom limb pain prior to smart prosthetic fitting.

## Data availability statement

The raw data supporting the conclusions of this article will be made available by the authors, without undue reservation.

## Ethics statement

The studies involving human participants were reviewed and approved by the Ethics Committee of Laboratory Animals of National Research Center of Rehabilitation for Technical Aids. The patients/participants provided their written informed consent to participate in this study.

## Author contributions

XS and YZ provided overall project supervision, helped to analyze and interpret the data, and assisted in writing the manuscript. SL and CW performed the experiments. SL and WF analyzed the data and result. SL wrote the manuscript. NC and FM had proofread the formatting. All authors helped to prepare the manuscript and approved the submitted version.
